# The Role of Vitamin D in Thyroid Diseases

**DOI:** 10.3390/ijms18091949

**Published:** 2017-09-12

**Authors:** Dohee Kim

**Affiliations:** 1 Division of Endocrinology, Department of Internal Medicine, Dankook University College of Medicine, Dandae-ro, Dongnam-gu, Cheonan-si, Chungnam 330-714, Korea; dh9070@hanmail.net or dh9070@dankook.ac.kr; Tel.: +82-041-550-3934; Fax: +82-041-556-3256; 2 Department of Kinesiologic Medical Science, Graduate, Dankook University, Cheonan 330-714, Korea

**Keywords:** autoimmune thyroid disease, Graves’ disease, Hashimoto’s thyroiditis, thyroid cancer, vitamin D

## Abstract

The main role of vitamin D is regulating bone metabolism and calcium and phosphorus homeostasis. Over the past few decades, the importance of vitamin D in non-skeletal actions has been studied, including the role of vitamin D in autoimmune diseases, metabolic syndromes, cardiovascular disease, cancers, and all-cause mortality. Recent evidence has demonstrated an association between low vitamin D status and autoimmune thyroid diseases such as Hashimoto’s thyroiditis and Graves’ disease, and impaired vitamin D signaling has been reported in thyroid cancers. This review will focus on recent data on the possible role of vitamin D in thyroid diseases, including autoimmune thyroid diseases and thyroid cancers.

## 1. Introduction

Vitamin D is a steroid molecule, mainly produced in the skin, which regulates the expression of a large number of genes [1]. The vitamin D receptor (VDR) is found in most tissues and cells in the body. The main role of vitamin D is regulating bone metabolism and calcium and phosphorus homeostasis. Recent evidence suggests that vitamin D deficiency, which is common worldwide, could also have non-skeletal actions including an important role in autoimmune diseases, cancers, metabolic syndromes, cardiovascular disease, and infection, as well as all-cause mortality [1,2,3]. Low levels of vitamin D have also been associated with autoimmune thyroid diseases (AITD) such as Hashimoto’s thyroiditis (HT) and Graves’ disease (GD). Impaired vitamin D signaling has been reported to encourage thyroid tumorigenesis [4,5,6].

The aim of this review is to summarize recent data on the possible association between vitamin D and thyroid diseases, including AITD and thyroid cancers.

This review is based on an electronic search of literature in the PubMed database published from January 2002 up to May 2017 using combinations of the following keywords: vitamin D or vitamin D deficiency/insufficiency and autoimmune thyroid disease, Hashimoto’s thyroiditis, Graves’ disease, thyroid cancer, or thyroid nodule. The references included in previously published review articles were also scanned, and any relevant papers were also included.

## 2. Vitamin D Sources, Metabolism, and Action

There are two forms of vitamin D, vitamin D_3_ (or cholecalciferol) and vitamin D_2_ (or ergocalciferol). The former is mainly synthesized in the skin by 7-dehydrocholesterol reductase upon exposure to ultraviolet B (UVB) radiation, and can be also obtained from a few dietary sources (mainly fatty fish), while the latter is produced by plants and fungi [7,8].

Both forms of vitamin D are transported to the liver where they are converted to 25-hydroxyvitamin D (25(OH)D or calcidiol) by 25-hydroxylase (CYP27A1 and CYP2R1). 25(OH)D is the major circulating and stored form of vitamin D, and the serum levels of this form are considered the best marker to measure whole body vitamin D status [5,7,8,9]. Although debated, vitamin D deficiency is usually defined as 25(OH)D level < 50 nmol/L, and vitamin D insufficiency as 50–75 nmol/L of 25(OH)D [1,2,10]. At physiological concentrations, 25(OH)D is biologically inactive and must be converted to the biologically active form 1,25-dihydroxyvitamin D (1,25(OH)_2_D or calcitriol) by 1α-hydroxylase (CYP27B1) in the kidneys. The activity of the 1α-hydroxylase enzyme is under strict control by parathyroid hormone (PTH), and is inhibited by high levels of 1,25(OH)_2_D and fibroblast growth factor 23 (FGF23). In addition, 1,25(OH)_2_D is inactivated by 24-hydroxylase (CYP24A1) [5,7,8,9]. However, other cell types, including immune cells, also express 1α-hydroxylase and are able to convert the inactive 25(OH)D into the active 1,25(OH)_2_D in either an autocrine or paracrine manner without the above feedback regulation [8]. It has been proposed that serum 25(OH)D levels serve as the main determining factor of extrarenal 1,25(OH)_2_D synthesis. Indeed, numerous associations have been found between vitamin D status (reflected by serum 25(OH)D concentration) and extraskeletal health outcomes, rather than with serum 1,25(OH)_2_D concentrations [5]. Vitamin D can be stored in and released from fat cells, and it is bound to the vitamin D binding protein (DBP). Approximately 88% of 25(OH)D and 85% of 1,25(OH)_2_D is bound by DBP, and 12–15% of circulating vitamin D is bound to albumin, with the free form having greater accessibility to target cells [5].

The active 1,25(OH)_2_D form binds to the nuclear vitamin D receptor (VDR), which acts on the vitamin D response element (VDRE) of target genes to exert its effects [5]. Directly and indirectly, 1,25(OH)_2_D controls the expression of over 200 genes, including genes responsible for the regulation of cellular proliferation, differentiation, apoptosis, and angiogenesis [2]. Among others, the brain, prostate, breast, and colon tissues, as well as immune cells, all express VDR and respond to 1,25(OH)_2_D. 1,25(OH)_2_D decreases the cellular proliferation of both normal cells and cancer cells and induces their terminal differentiation, in addition to being a potent immunomodulator [2]. A membrane-bound VDR may also exist, which would mediate more immediate, non-genomic actions of 1,25(OH)_2_D [9].

Recently, several genetic studies have demonstrated that an individual’s susceptibility to autoimmune disorders and cancers is associated with polymorphisms in numerous proteins and enzymes associated with vitamin D function, including VDR, DBP, CYP27B1, CYP2R1, and CYP24A1. Four single-nucleotide polymorphic (SNP) variants of the *VDR* gene (*ApaI*, *BsmI*, *FokI*, and *TaqI*) are most frequently studied [5,11].

## 3. Vitamin D and Autoimmune Thyroid Diseases

### 3.1. Mechanisms

Autoimmune thyroid diseases, including HT and GD, are the most common organ-specific autoimmune disorders [12]. These AITDs are polygenic diseases resulting from a combination of genetic predisposition (thyroid-specific genes and immune-modulating genes) and environmental triggers (iodine, selenium, drugs, irradiation, smoking, infections, stress, etc.), characterized by lymphocytic infiltration into the thyroid gland and production of thyroid-specific autoantibodies [12,13]. Chronic autoimmune thyroiditis, or HT, is a typical T-cell-mediated autoimmune disease characterized by a diffuse goiter, the presence of anti-thyroid peroxidase (anti-TPO) and/or anti-thyroglobulin (anti-Tg) antibodies in serum, varying degree of thyroid hypofunction, and intrathyroidal infiltration of B and T lymphocytes with CD4^+^ type 1 T helper (Th1) subtype predominance. In GD, lymphocytic infiltration is mild and involves mainly CD4^+^ type 2 T helper (Th2) cells, which induce the production of antibodies to bind to the thyroid stimulating hormone (TSH) receptor. This stimulates the growth and function of thyroid follicular cells leading to hyperthyroidism, indicating a humoral immune response [12]. In summary, in genetically predisposed individuals, the disruption of these immune-endocrine interactions by environmental factors is able to shift the balance between Th1-Th2 immune response. This results in a Th1-cell-mediated autoimmune reaction with thyrocyte destruction and hypothyroidism in HT, but in a hyperreactive Th2-mediated humoral response against TSH receptor (TSHR) with stimulatory antibodies leading to hyperthyroidism in GD [12].

Vitamin D plays a significant role in modulation of the immune system, enhancing the innate immune response while exerting an inhibitory action on the adaptive immune system [7,11]. Most immune cells, including T cells, B cells, and antigen-presenting cells (APCs), such as dendritic cells (DCs) and macrophages, express VDR and 1α-hydroxylase [7,8,14]. At the level of the APCs, 1,25(OH)_2_D inhibits the surface expression of major histocompatibility complex class II antigens and co-stimulatory molecules, and prevents the differentiation and maturation of DCs as well as their activation and survival, leading to decreased antigen presentation and T cell activation. Moreover, 1,25(OH)_2_D also modulates DC-derived cytokine expression by inhibiting the production of interleukin (IL)-12 and IL-23 (major cytokines driving Th1 differentiation and IL-17 producing T helper (Th17) cell differentiation, respectively) and enhancing the release of IL-10. Thereby, 1,25(OH)_2_D indirectly shifts the polarization of T cells from a Th1 and Th17 phenotype towards a Th2 phenotype [14,15]. In addition, 1,25(OH)_2_D directly leads to a shift from a pro-inflammatory to a more tolerogenic immune status, which includes diverse effects on T cell subtypes. 1,25(OH)_2_D inhibits Th1 cell proliferation, differentiation, and production of cytokines (IL-2 and interferon-γ), as well as Th17-derived cytokines (IL-17 and IL-21), but also promotes the production of anti-inflammatory Th2 cytokines (IL-3, IL-4, IL-5, and IL-10), shifting the balance from a Th1 and Th17 phenotype to a Th2 cell phenotype. 1,25(OH)_2_D favors regulatory T cell (Treg) cell development via modulation of DCs and by directly targeting T cells, thereby blocking Th1 development. Finally, 1,25(OH)_2_D inhibits B cell proliferation and differentiation into plasma cells, immunoglobulin secretion (IgG and IgM), memory B cell generation, and also induces B cell apoptosis [7,8,11,14,15,16]. The ability of 1,25(OH)_2_D to suppress the adaptive immune system promotes immune tolerance and appears to be beneficial for a number of autoimmune disease [7,8].

### 3.2. Animal Studies

The administration of 1,25(OH)_2_D in addition to cyclosporine was demonstrated to effectively prevent the induction of experimental autoimmune thyroiditis (EAT) with a synergistic effect in CBA mice [17,18]. In another study using an EAT model in Wistar rats, 1,25(OH)_2_D prevented and ameliorated the pathological changes of the thyroid gland and corrected thyroid autoantibody production and the cytokine disequilibrium [19]. In addition, vitamin D-deficient BALB/c mice developed persistent hyperthyroidism after immunization with TSHR, not seen in vitamin D-sufficient mice [20].

### 3.3. Human Studies

Several clinical studies have reported a low vitamin D status in AITD or HT, indicating an association between vitamin D deficiency and thyroid autoimmunity (Table 1) [21,22,23,24,25,26,27,28,29,30,31,32,33,34]. Kivity et al., reported that the prevalence of vitamin D deficiency (25(OH)D level < 25 nmol/L) was significantly higher in 50 patients with AITD compared with 98 healthy individuals (72% vs. 30.6%; *p* < 0.001) as well as in 28 patients with HT compared to 42 patients with non-AITD (79% vs. 52%; *p* < 0.05). Vitamin D deficiency was also found to be correlated with the presence of anti-thyroid antibodies (*p* = 0.01), suggesting the involvement of vitamin D in the pathogenesis of AITD [21]. Tamer et al., demonstrated that the prevalence of vitamin D insufficiency (25(OH)D level < 75 nmol/L) in 161 HT cases was significantly higher than in 162 healthy controls (92% vs. 63%; *p* < 0.0001). Among the HT cases, the prevalence of vitamin D insufficiency tended to be higher in patients with overt hypothyroidism (47/50, 94%) or subclinical hypothyroidism (44/45, 98%) than in those with euthyroidism (57/66, 86%), although the differences were not statistically significant [22]. Bozkurt et al., revealed that serum 25(OH)D levels of HT patients (180 treated and 180 non-treated) were significantly lower than 180 controls, and that the severity of vitamin D deficiency was correlated with the duration of HT, thyroid volume, and antibody levels, suggesting a potential role of vitamin D in the development of HT and/or its progression to hypothyroidism [23]. By comparing 41 hypothyroid HT patients with 45 healthy euthyroid individuals, Mansournia et al., found a significant inverse association between serum 25(OH)D levels and HT (OR 0.81, 95% CI 0.68–0.96; *p* = 0.018), such that each 12.5 nmol/L increase in serum 25(OH)D level resulted in a 19% decrease in the odds of HT [24]. Shin et al., reported that 111 patients with elevated anti-thyroid antibodies had lower levels of serum 25(OH)D_3_ than 193 patients with no elevation (*p* < 0.001). Moreover, after adjusting for age, sex, and body mass index (BMI), a negative correlation (*r* = −0.252; *p* < 0.001) was found between 25(OH)D_3_ and anti-TPO levels in a group of individuals with AITD [25]. Unal et al., demonstrated that 254 newly diagnosed HT and 27 GD patients had lower 25(OH)D levels than 124 healthy controls (*p* < 0.001), and serum 25(OH)D levels were inversely correlated with anti-Tg (*r* = −0.136; *p* = 0.025) and anti-TPO (*r* = −0.176; *p* = 0.003) antibodies [26]. Choi et al., analyzed 6685 subjects who underwent routine health checkups and found significantly lower serum 25(OH)D levels in pre-menopausal women with AITD, but not in postmenopausal women. Vitamin D deficiency (<25 nmol/L) and insufficiency (25–75 nmol/L) were significantly associated with AITD only in pre-menopausal women, suggesting a possible link between vitamin D and estrogen in the development of AITD [27]. In a population-based health survey including 1714 Chinese adults, Wang et al., also showed a negative correlation (*r* = −0.121; *p* = 0.014) between 25(OH)D and anti-Tg levels, but only in female subjects [28]. Recently, the author reported that the prevalence of vitamin D insufficiency (25(OH)D level < 75 nmol/L) was significantly higher in 369 AITD patients (221 HT and 148 GD) than in 407 non-AITD patients (*p* = 0.011), and was higher in HT patients than in those with GD or non-AITD (*p* = 0.017). In addition, among the HT cases, patients with overt hypothyroidism had a higher prevalence of vitamin D insufficiency (*p* < 0.001) and lower 25(OH)D levels (*p* = 0.009) compared with HT patients with euthyroidism and subclinical hypothyroidism or patients without AITD. Serum 25(OH)D levels were significantly negatively correlated with serum TSH levels after adjustment for age, sex, BMI, and sampling season (*r* = −0.127; *p* = 0.013) [29]. A recent meta-analysis of 20 case-control studies showed that AITD patients have lower 25(OH)D levels and are more likely to be vitamin D deficient compared to controls [30]. Subgroup analyses showed that GD and HT patients also have lower 25(OH)D levels and are more likely to be deficient in vitamin D. The criterion for vitamin D deficiency in the studies included in this meta-analysis was a 25(OH)D level of <25–50 nmol/L [30]. In addition, Muscogiuri et al., studied 168 elderly subjects (mean age of 82 years) and showed a significantly higher prevalence of AITD in subjects with vitamin D deficiency (25(OH)D level < 50 nmol/L) (*p* = 0.002) and a significant correlation between 25(OH)D and anti-TPO levels (*r* = −0.27; *p* = 0.03) [31].

A few studies have focused on investigating the correlation between low vitamin D status and HT in children. Camurdan et al., observed higher rates of vitamin D deficiency (25(OH)D level < 37.5 nmol/L; 73.1% vs. 17.6%; *p* < 0.0001) and lower 25(OH)D levels (31.2 vs. 57.9 nmol/L; *p* < 0.001) in 78 children with recently diagnosed HT compared with 74 controls, and an inverse correlation between 25(OH)D and anti-TPO levels (*r* = −0.30; *p* = 0.007) [32]. Evliyaoğlu et al., studied 90 children with HT and 79 age-matched healthy controls. The prevalence of vitamin D deficiency (25(OH)D level < 50 nmol/L) in HT patients was significantly higher than in the control group (71.1% vs. 51.9%; *p* = 0.025). The mean serum 25(OH)D_3_ level in the HT group was significantly lower compared to the control group (41.6 vs. 52.4 nmol/L; *p* = 0.001), and HT was observed 2.28 times more frequently in individuals with 25(OH)D levels of <50 nmol/L (OR 2.28, 95% CI 1.21–4.3) [33]. Metwalley et al., investigated 56 children with autoimmune thyroiditis (AIT) and 56 healthy controls and reported similar results, including a higher rate of vitamin D deficiency (71.4% vs. 21.4%; *p* < 0.001) and lower 25(OH)D levels (16.2 vs. 33.9 nmol/L; *p* < 0.001) in the AIT group compared to the controls. The difference was more evident in patients with overt hypothyroidism than those with subclinical hypothyroidism (9.5 vs. 16.4 nmol/L; *p* < 0.01). In addition, there were significant negative correlations between serum 25(OH)D and patient age, disease duration, BMI, and levels of anti-TPO, anti-Tg and TSH (*p* < 0.001 each). The authors proposed that low serum vitamin D is significantly associated with AIT, but is not an independent risk factor for the progression of AIT to overt hypothyroidism [34].

Of note, a few studies that have evaluated whether vitamin D supplementation is beneficial for AITD or HT have been published recently. Mazokopakis et al., showed that serum 25(OH)D levels in 218 euthyroid HT patients were inversely correlated with anti-TPO levels. Anti-TPO levels were also significantly higher in vitamin D-deficient HT patients (25(OH)D level < 75 nmol/L) compared to HT patients with no vitamin D deficiency. After 4 months of oral vitamin D_3_ supplementation (1200–4000 IU/day) in 186 vitamin D-deficient patients, there was a significant decrease (20.3%) in serum anti-TPO levels [41]. Chaudhary et al., analyzed 100 newly diagnosed AITD patients and found that anti-TPO levels were highest among patients in the lowest 25(OH)D quartile (*p* = 0.084). At the 3-month follow-up, there was a significant fall in anti-TPO levels in patients that received vitamin D_3_ supplementation at 60,000 IU per week for 8 weeks when compared to patients without vitamin D supplementation (−46.73% vs. −16.6%; *p* = 0.028). The number of responders (≥25% fall in anti-TPO level) was higher in the group that received vitamin D supplementation (68% vs. 44%; *p* = 0.015) [42]. Simsek et al., demonstrated that anti-TPO and anti-Tg levels in AITD patients with a 25(OH)D level <50 nmol/L were significantly decreased as a result of administration of vitamin D at 1000 IU/day for 1 month (*p* = 0.02 and *p* = 0.03, respectively) [43].

Some studies, however, have failed to find an association between low vitamin D status and AITD or HT. Goswami et al., revealed no association of vitamin D deficiency (<25 nmol/L) and anti-TPO positivity, but only a weak inverse correlation between serum 25(OH)D and anti-TPO levels in 642 students, teachers, and staff from India (*r* = −0.08; *p* = 0.04) [35]. A total of 803 subjects from an AITD cohort from Amsterdam were investigated in a longitudinal study by Effraimidis et al., who showed that 25(OH)D levels were not lower in AIRD cases compared to controls, nor in subjects with a genetic susceptibility for AITD or seroconverters with de novo occurrence of anti-TPO antibodies. The authors concluded that vitamin D deficiency is not associated with the early stages of thyroid autoimmunity [36]. D’Aurizio et al., also reported no differences in vitamin D deficiency (<50 nmol/L) and 25(OH)D levels between 100 AITD patients (52 HT and 48 GD) and 126 healthy controls [11]. In contrast to the above results, Yasmeh et al., investigated 97 HT patients and 88 healthy controls and reported a higher 25(OH)D level and higher rate of vitamin D sufficiency (≥75 nmol/L) in HT females relative to control females, but there were no differences in males. A significant positive correlation between 25(OH)D and anti-TPO levels was observed in males (*r* = 0.436; *p* = 0.016). The authors pointed out that HT was not associated with higher rates of vitamin D deficiency relative to the control group [37].

A relationship between vitamin D and GD has also been reported. In a study by Kivity et al., the prevalence of vitamin D deficiency in GD was found to be higher than in controls, but was not significantly different compared to that in non-AITD patients [21]. Other authors have reported that vitamin D levels are no lower in patients with GD than in healthy controls [11] or in non-AITD patients [29]. In contrast, Yasuda et al., found that the serum 25(OH) levels of 26 female patients with new-onset GD were lower (35.9 vs. 42.7 nmol/L; *p* < 0.05) and the prevalence of vitamin D deficiency (25(OH)D level < 37.5 nmol/L) was higher (65.4% vs. 32.4%; *p* = 0.05) than that recorded in 46 controls. There was a significant association between serum 25(OH)D_3_ levels and thyroid volume (*r* = −0.45; *p* = 0.05) [38]. The same research group also reported that serum vitamin D levels were lower in 36 female patients without GD remission compared with 18 patients that had achieved GD remission (*p* < 0.005) and 49 controls (*p* < 0.0005) after 1 year [39]. Zhang et al., revealed that serum vitamin D levels in anti-TSHR antibody-positive GD patients were lower than in healthy controls or anti-TSHR antibody-negative patients, and were inversely correlated with anti-TSHR antibody levels, suggesting a possible link between vitamin D status and increased thyroid autoimmunity in GD patients [40]. In addition to the aforementioned meta-analysis, another meta-analysis of 26 studies showed that patients with GD had a higher prevalence of vitamin D deficiency compared to controls with high heterogeneity [44].

Several authors have studied the association between *VDR* gene polymorphisms (*ApaI*, *BsmI*, *FokI*, and *TaqI*) and AITD risk, although the results are still ambiguous and inconsistent. The first meta-analysis to assess the association between *VDR* gene polymorphisms and GD comprised seven studies (three Caucasian and four Asian). *ApaI*, *BsmI*, and *FokI* polymorphisms in the *VDR* gene were associated with susceptibility to GD in Asian populations, whereas there was no association between *VDR* gene polymorphisms and GD in Caucasian populations [45]. In a recent meta-analysis including eight studies (five European, two Asian, and one African), the *VDR BsmI* or *TaqI* polymorphisms were found to be significantly associated with AITD risk, including HT and GD (OR 0.801 for B vs. b; OR 0.854 for t vs. T), while the *ApaI* and *FokI* polymorphisms were not. Subgroup analysis of the European studies showed a decreased risk of AITD for the B or t variants [46]. Later, Inoue et al., demonstrated that the frequency of the TT genotype for *TaqI* was higher in GD patients than in HT patients, and the frequency of the C allele for *ApaI* was higher in GD patients than in controls. The frequency of the CC genotype for the *FokI* polymorphism was also higher in HT patients than in control subjects and GD patients. In addition, they reported that the genetic differences in the *DBP* and *CYP2R1* genes might be involved with the intractability of GD [47]. Wang et al., published a meta-analysis of 11 studies (five Caucasian and six Asian) in which the *VDR FokI* polymorphism was found to be associated with HT risk only in the Asian population (F vs. f; OR = 1.45) but not in the Caucasian population. Furthermore, the *TaqI*, *ApaI*, and *BsmI* polymorphisms were not positively associated in the overall population, nor when stratified by ethnicity [48].

Pani et al., investigated the association of *DBP*-gene polymorphisms and thyroid autoimmunity in 187 Caucasian families (561 participants). An intron 8 (TAAA)(n) polymorphism was found to be correlated with GD but not with HT [49]. However, a *CYP27B1* intron 6 polymorphism was not associated with GD or HT, as identified by the same authors [50]. In another study analyzing 332 polish patients with GD and 185 healthy controls, the *DBP* gene Lys allele at codon 420 was found to be associated with increased susceptibility to GD [51].

In conclusion, most studies have shown an association between low vitamin D status in the pathogenesis of AITD, especially HT. However, there are only few preliminary interventional studies for HT. Further randomized controlled trials are needed to determine whether there is a causal relationship, and investigate the potential application of vitamin D in the treatment of AITD.

## 4. Vitamin D and Thyroid Cancers

### 4.1. Mechanisms

In addition to its main role in calcium homeostasis, vitamin D can directly or indirectly regulate multiple signaling pathways involved in cellular proliferation, differentiation, apoptosis, inflammation, invasion, angiogenesis, and metastasis. Therefore, it has the potential to affect cancer development and growth. Recent findings indicate that vitamin D also regulates microRNA expression and may influence cancer stem cell biology [52].

Both in vitro models and in vivo animal models have demonstrated that vitamin D has anti-proliferative, pro-differentiative, pro-apoptotic, and anti-inflammatory actions within the tumor microenvironment. Perhaps the most recognized anti-neoplastic effect of calcitriol is its ability to inhibit cell proliferation [53]. 1,25(OH)_2_D increases the expression of the cyclin-dependent kinase (CDK) inhibitors p21 and p27, and decreases CDK activity, thereby leading to dephosphorylation of the retinoblastoma (Rb) protein and G0/G1 cell cycle arrest. 1,25(OH)_2_D has indirect effects on cell cycle regulation through inhibition of mitogenic signaling by growth factors such as insulin-like growth factor 1 (IGF1) and epidermal growth factor (EGF), as well as by inducing increased expression of growth inhibitors such as transforming growth factor-β (TGF-β). In addition, 1,25(OH)_2_D modulates intracellular kinase pathways such as p38, MAPK, ERK, and PI3K, represses the proto-oncogene *MYC*, and inhibits the high telomerase activity characteristic of human cancer cells by decreasing telomerase reverse transcriptase (*TERT*) mRNA expression [52,53,54]. Besides its anti-proliferative effects, 1,25(OH)_2_D also regulates key mediators of apoptosis such as inhibiting anti-apoptotic proteins (BCL-2 and BCL-X_L_) or inducing the expression of pro-apoptotic proteins (BAX, BAK, and BAD). Moreover, 1,25(OH)_2_D induces caspase activation [53,54]. 1,25(OH)_2_D is a well-known pro-differentiation hormone that regulates the activity of more than 60 genes involved in cell differentiation [53]. Cell type-specific pro-differentiation mechanisms include the regulation of β-catenin, JUN N-terminal kinase, PI3K, and nuclear factor-κB (NF-κB) signaling pathways, as well as regulating the activity of transcription factors such as the activator protein 1 complex and CCAAT/enhancer-binding protein (C/EBP) [52]. 1,25(OH)_2_D shows beneficial anti-inflammatory actions in several cancers through key mechanisms including the inhibition of prostaglandin synthesis (by suppressing cyclooxygenase 2 (COX2) expression) and prostaglandin signaling (by upregulating the expression of the catabolic enzyme and repressing the expression of prostaglandin receptors), suppressing p38 stress kinase signaling and subsequent inhibition of the production of pro-inflammatory cytokines, and the inhibition of NF-κB signaling [52,53]. In addition, 1,25(OH)_2_D inhibits invasion and metastasis by regulating the expression of components of the plasminogen activator system and matrix metalloproteinases (MMPs) and increasing the expression of E-cadherin, a tumor suppressor gene that is inversely correlated with metastatic potential. 1,25(OH)_2_D also inhibits angiogenesis by suppressing the expression of vascular endothelial growth factor (VEGF) [52,53].

Thyroid cancer is the most common endocrine malignancy worldwide, and its incidence is gradually increasing. Risk factors such as exposure to ionizing radiation, chemical genotoxins, and obesity have been implicated in the increasing incidence of thyroid cancer [55]. The molecular pathways involved in thyroid carcinogenesis are becoming increasingly unraveled. Thyroid neoplasia results from genomic instability that is enhanced by risk factors such as exposure to radiation resulting in early genetic alterations (e.g., *RET/PTC* and *PAX8-PPARγ* rearrangements, *BRAF* and *RAS* point mutations) and constitutive activation of thyrocyte signaling pathways. Dedifferentiation is caused by late genetic alterations (e.g., *p*53 and *CTNNB1* mutations), resulting in rapidly expanding and metastatic thyroid cancer [54].

Several preclinical studies have demonstrated growth arrest of thyroid cancer after the administration of pharmacological doses of 1,25(OH)_2_D or its analogs in differentiated thyroid cancer (DTC) cell lines. The mechanism underlying the antitumor effect of 1,25(OH)_2_D on thyroid cancers were mainly found to result from inhibited proliferation, although the effects of redifferentiation and apoptosis were not consistent [54]. Specifically, it has been shown that 1,25(OH)_2_D inhibits cell proliferation by inhibiting the expression of *c-MYC*, a well-known proto-oncogene, and stimulates the accumulation of *p*27, resulting in an increased percentage of cells in the G0-G1 phase of the cell cycle with decreased *Ki67* expression. In addition to this, 1,25(OH)_2_D has also been found to increase cell adhesiveness via increased fibronectin expression, mediated by the PTEN/PI3 kinase pathway [54,55].

### 4.2. In Vitro and Animal Studies

In vitro, 1,25(OH)_2_D and a new less-calcemic vitamin D analog, MART-10, could effectively repress ATC cell migration and invasion through reversing cadherin switch, blocking EMT processes, and downregulating the formation of F-actin. As MART-10 shows more potent attenuation of ATC cell metastatic potential than 1,25(OH)_2_D, and considering that MART-10 has been confirmed to be active in vivo without inducing any obvious side effects, further in vivo study of the applicability of MART-10 for ATC treatment is warranted [56]. In another study, 1,25(OH)_2_D was shown to inhibit the proliferation of thyroid cancer stem cells derived from ATC via G2/M phase cell cycle arrest [57].

In female SCID mice that were implanted with human thyroid follicular carcinoma-derived (WRO) cells, calcitriol administration was observed to reduce tumor size, enhance cellular differentiation, and prevent metastatic growth as indicated by marked p27 accumulation in the thyroid carcinoma cells [58]. In other study using the same mouse model, treatment with 1,25(OH)_2_D and its noncalciomimetic analog EB1089 resulted in a 50% reduction in tumor weight through increased cell adhesiveness accompanied by an increase in fibronectin [59].

### 4.3. Human Studies

Multiple cell culture and animal models of cancer support a role for dietary vitamin D_3_ and calcitriol in impeding cancer development and progression. However, data from human clinical trials are inconsistent [52]. A large number of epidemiological studies have suggested that low circulating levels of 25(OH)D, related to geographical location, diet, and outdoor activity, are associated with a higher risk of cancer incidence and poorer outcomes, although many studies do not demonstrate any associations [52,53]. A recent review of epidemiological evidence of the relationship between vitamin D and colorectal, breast, and prostate cancers reported a consistent inverse association for colorectal cancer incidence and mortality in observational studies, whereas the results for breast cancer have generally shown that higher 25(OH)D levels are associated with a lower risk of progression and mortality [60]. In contrast, the randomized, double-blind clinical trials conducted to date have generally failed to support these findings. For prostate cancer, there is no convincing evidence of an association between 25(OH)D and incidence, progression and mortality, although the results of one open-label clinical trial suggested that supplementation with 4000 IU/day of vitamin D_3_ may inhibit progression of the disease [60].

A few clinical studies have suggested an association between vitamin D status and incidence or worse outcomes of thyroid cancer (Table 2). Roskies et al., retrospectively studied preoperative 25(OH)D levels in 212 patients undergoing thyroidectomy, and showed a higher malignancy rate in the vitamin D-deficient group (25(OH)D level < 37.5 nmol/L) compared to the vitamin D-sufficient group (75% vs. 37.5%), corresponding to a relative risk of 2.0 (*p* = 0.03), suggesting that vitamin D deficiency is a potentially modifiable risk factor for thyroid cancer [61]. Sahin et al., reported that 344 patients with PTC had significantly lower 25(OH)D levels than 116 controls (42.4 vs. 47.4 nmol/L; *p* = 0.004), and vitamin D deficiency (25(OH)D level < 50 nmol/L) was more prevalent in this group (166/235 vs. 64/108; *p* = 0.026). The tumor diameter was also significantly associated with log-vitamin D_3_ concentrations (*B* = 0.207; *p* = 0.04) [62]. Kim et al., investigated 548 female patients who underwent total thyroidectomy for PTC. The preoperative 25(OH)D levels were significantly lower in patients with a tumor size of >1 cm (*p* = 0.041) or lymph node metastasis (*p* = 0.043). Patients with 25(OH)D levels < 46.2 nmol/L (median) also had a significantly higher risk of T stage 3/4, lymph node metastasis, and progressing to stage III/IV after adjustment [63]. Stepien et al., revealed significantly lower 1,25(OH)_2_D levels in 27 PTC patients, 16 FTC patients, and seven ATC patients compared to 26 healthy controls, and also reported an inverse relationship of lower 1,25(OH)_2_D levels with an advanced tumor stage. However, there were no significant differences in 25(OH)D levels [64]. Penna-Martinez et al., showed lower serum 1,25(OH)_2_D levels in 172 patients with DTC when compared to 321 healthy controls. While no significant difference in 25(OH)D levels were observed, there was an association between VDR polymorphisms and the incidence of thyroid carcinoma [65]. Later, the same authors investigated vitamin D status and expression of genes related to vitamin D enzymes in 253 patients with DTC and 302 controls, and they showed an association between DTC and low 25(OH)D and 1,25(OH)_2_D levels in certain CYP24A1 haplotypes [66].

Besides the aforementioned studies, contrasting data that suggest there is no association between 25(OH)D and thyroid cancer have been reported by other groups. Laney et al., demonstrated that serum 25(OH)D levels and the proportion of patients with 25(OH)D levels <75 nmol/L were not different in 42 cases with thyroid nodules, 45 with thyroid cancer in remission, and 24 active thyroid cancer patients, and these factors were not affected by cancer stage [67]. Jonklaas et al., revealed no associations between preoperative 25(OH)D levels and diagnosis of thyroid cancer, disease stage, or any other prognostic features in 65 euthyroid patients undergoing thyroidectomy for thyroid cancer [68]. Lizis-Kolus et al., demonstrated no difference in serum 25(OH)D levels in 40 female patients with PTC and 40 with HT, and found no relationship between 25(OH)D and the clinical disease stage of patients with PTC [69]. Ahn et al., showed that preoperative 25(OH)D levels were not associated with either disease aggressiveness or poor outcomes among 820 patients with PTC [70]. Danilovic et al., evaluated 433 patients that underwent thyroidectomy for thyroid nodules and observed no significant differences in preoperative 25(OH)D levels or quartile distribution between benign and malignant cases. Decreased 25(OH)D levels were not overtly associated with a poorer prognosis among 187 patients with PTC [71]. The author also investigated 410 patients that underwent ultrasonography-guided fine-needle aspiration for thyroid nodules and found that serum 25(OH)D levels and the prevalence of vitamin D insufficiency (25(OH)D level < 75 nmol/L) did not significantly differ between benign and malignant thyroid nodules. In addition, vitamin D insufficiency was not associated with disease stage or any other prognostic factors among 44 DTC patients [72]. More recently, Choi et al., analyzed 5186 euthyroid subjects with no clinical evidence of AITD who underwent routine health check-ups, and found that serum 25(OH)D levels were not associated with thyroid cancer prevalence [73].

No convincing associations have been shown for studies that have estimated vitamin D intake and related this to the prevalence of thyroid cancer [55]. In a population-based case-control study, weekly use of a vitamin D supplement was not related to thyroid cancer risk in females from Los Angeles [74]. In contrast, the cross-sectional cohort Vitamins and Lifestyle (VITAL) study showed a positive association between high dietary supplementation of vitamin D and thyroid cancer risk (OR 1.66, 95% CI 1.21–2.28) [75]. The large prospective US National Institutes of Health American Association of Retired Persons (NIH-AARP) Diet and Health Study showed no clear evidence of a positive or negative association between thyroid cancer risk and dietary intake of any micronutrient, including vitamin D [76]. A systematic review suggested that the current evidence to support any protective or hazardous effect of vitamin or mineral supplements, including vitamin D, on thyroid cancer development is inconclusive [77]. Vitamin D levels were not measured in these studies, and inconsistent data may be due to the general poor correlation between vitamin D deficiency and estimates of vitamin D intake [55].

The VDR is expressed in both normal and malignant thyroid tissue [54]. Moreover, VDR and CYP27B1 expressions were found to be increased in papillary thyroid cancer (PTC) when compared to normal thyroid tissue [78]. Later, these findings were reproduced by other groups which reported that VDR, CYP27B1, and CYP24A1 expressions were increased in follicular adenoma and DTC, such as PTC and follicular thyroid cancer (FTC), when compared to normal thyroid tissue [79]. When DTC was compared to follicular adenoma, significantly higher VDR and lower CYP24A1 expression was observed. The high VDR/low CYP24A1 profile in DTC might suggest a stronger antitumor response, increasing the local availability and reactivity of 1,25(OH)_2_D [54,79]. However, in PTC with lymph node metastasis, VDR and CYP24A1 were decreased compared with non-metastasized PTC, along with decreased expression of CDK inhibitors [79]. In anaplastic thyroid cancer (ATC), VDR expression is often lost. Moreover, ATC with high Ki67 expression or distant metastases at diagnosis was characterized by more negative VDR/CYP24A1/CYP27B1 staining [79]. In conclusion, increased expression of key players involved in local 1,25(OH)_2_D signaling was demonstrated in benign and differentiated malignant thyroid tumors, but a decrease or complete loss of expression was observed for local nodal and especially distant metastasis of ATC, suggesting a local antitumor response of 1,25(OH)_2_D in the early stages of cancer [79]. In addition, the mRNA expression of VDR and genes regulating cell–cell and cell–matrix adhesion and degradation of the extracellular matrix (ECM), ECM1 and type II transmembrane serine protease-4 (TMPRSS4) were higher in PTC samples compared to normal thyroid tissue from the same patients. Increased VDR expression was often linked to increased expression of ECM1 and/or TPMRSS4, both of which are potential markers of PTC. ECM1 promotes blood vessel formation, stimulating the proliferation of malignant epithelial cells and leading to tumor progression, and TMPRSS4 modulates invasion, metastasis, migration, and adhesion, as well as epithelial–mesenchymal transition (EMT) in cancer cells. Up-regulation of ECM1 or PMPRSS4 has been reported in numerous malignant epithelial tumors, and is an independent predictor of a thyroid carcinoma [80]. In addition to being elevated in PTC, VDR mRNA overexpression was recently reported to be correlated with worse prognostic factors, such as subtypes of PTC with a poorer prognosis, lateral neck node metastasis, advanced stage, and recurrence-free survival [81].

Significant associations with *VDR* polymorphisms have been reported for prostate (*Fok1*, *Bsm1*, and Taq1), breast (Fok1, Bsm1, and *Apa1*), colon-rectum (*Fok1*, *Bsm1*, and *Taq1*), and skin (*Fok1*, *Bsm1*, and *Taq1*) cancers [82]. However, only two studies have investigated the association between *VDR* polymorphisms and thyroid cancer risk. One showed no difference in *VDR* polymorphisms between 71 DTC patients and 82 healthy controls [83]. The other revealed that while the AA and FF alleles of the *ApaI* and *FokI VDR* polymorphisms and the tABF haplotype confer protection against FTC, the Tabf haplotype appeared to be associated with an increased FTC risk in 172 DTC patients and 321 healthy controls [65].

In summary, although many preclinical studies suggest an association between vitamin D and thyroid cancer, only a few conflicting case-control studies have been reported. To date, no interventional, randomized controlled trials have been published, and further studies are needed.

## 5. Limitations in the Study of Vitamin D

Although many studies have suggested an association of low vitamin D status with AITD and thyroid cancer, epidemiological studies simply show correlative relationships which cannot be used to determine cause and effect [3,72]. Low vitamin D status may be not the cause but rather a consequence of the disease [41,84]. The low vitamin D levels of patients with thyroid diseases can be explained by low vitamin D intake, malabsorption, lack of sun exposure, or reduced outdoor activity [21,29,72,84,85]. In addition, it is likely that good vitamin D status represents a general marker of good health. A young individual with a normal body weight and a healthy lifestyle, including good dietary and exercise habits, is more likely to not only have higher 25(OH)D levels, but also a lower risk of cancer or chronic illness. Therefore, it is difficult to separate the effects of these characteristics from those that may be attributed to 25(OH)D levels [55,76,86]. Similarly, low vitamin D status may be the result of chronic illness, which prevents outdoor activities and sun exposure. Vitamin D is rarely ingested in isolation; therefore, additional nutrients that are co-ingested with vitamin D may have independent or synergistic effects [86]. Additionally, because serum vitamin D levels were measured only once in almost all observational studies, the values obtained might not be representative [70,72,86].

The controversial and varying results of studies are partly due to inter-assay and inter-laboratory variability in the measurements of 25(OH)D, seasonal variations in blood sampling of 25(OH)D, and the different cut-off levels used to define vitamin D deficiency or insufficiency. In addition, the conflicting results could be explained by limitations in study design, such as cross-sectional studies with a small number of subjects, and therefore, the potential for selection bias, as well as the heterogeneity of the study population and the diverse methods used for the diagnosis of AITD, HT, GD, or thyroid cancer [11,29,41,72]

## 6. Conclusions

The pleiotropic roles of vitamin D have been recognized through preclinical and observational studies which have suggested a beneficial role of vitamin D in the management of thyroid disease. However, only an ambiguous causal relationship and few interventional studies have been reported to date, so the preventive and therapeutic potential of vitamin D or its analog in thyroid diseases remains debated. Ongoing and future long-term, randomized controlled trials are required to determine whether individuals with low 25(OH)D levels are at increased risk of developing AITD and thyroid cancer, and to provide insight into the efficacy and safety of vitamin D as a therapeutic tool for these thyroid diseases.

## Figures and Tables

**Table 1:** Clinical studies for an association between vitamin D status and autoimmune thyroid diseases.

Sources	Study Subjects	Low Vitamin D Status (25(OH)D Level (nmol/L))	
		Criteria	Notable Findings
Kivity et al., 2011 [21]	50 AITD (28 HT, 22 GD), 42 non-AITD, 98 healthy controls	<25	70% of HT, 64% of GD, 52% of non-AITD patients, 30% of controls (SD)
Tamer et al., 2011 [22]	161 HT, 162 healthy controls	<75	91.9% of HT, 63% of controls (SD)
Bozkurt et al., 2013 [23]	180 euthyroid HT, 180 newly diagnosed HT, 180 healthy controls	<25	48.3% vs. 35% vs. 20.5% of each groups (SD); correlated with thyroid volume (*r* = 0.15), anti-TPO (*r* = −0.36), anti-Tg levels (*r* = −0.34) (SD)
Mansournia et al., 2014 [24]	41 hypothyroid HT, 45 healthy controls	NA	inverse association with HT (OR 0.81 for 12.5 nmol/L increase in 25(OH)D) (SD)
Shin et al., 2014 [25]	111 AITD, 193 non-AITD patients	NA	31.5 nmol/L in AITD, 36.2 nmol/L in non-AITD (SD); negative correlation between 25(OH)D and anti-TPO levels (*r* = −0.252) (SD)
Unal et al., 2014 [26]	254 newly diagnosed HT, 27 GD, 124 healthy controls	NA	37.2 vs. 48.4 vs. 56.2 nmol/L in each groups (SD); correlated with anti-Tg (*r* = −0.14), anti-TPO levels (*r* = −0.18) (SD)
Choi et al., 2014 [27]	673 anti-TPO (+), 6012 anti-TPO (−) subjects for routine health checkups	<25 (D) 25–75 (I) >75 (S)	50.7 nmol/L in anti-TPO (+), 56.4 nmol/L in anti-TPO (−) in premenopausal women (SD); anti-TPO (+) 21.2%, 15.5.%, and 12.6% in D, I, S groups in premenopausal women (SD); OR 1.95 for TPO-Ab (+) (SD)
Wang et al., 2015 [28]	1714 subjects for population-based health survey	NA	correlation (*r* = −0.12) between 25(OH)D and anti-Tg levels only in female subjects (SD)
Kim, 2016 [29]	369 AITD (221 HT. 148 GD), 407 non-AITD patients	<75	46.1% of AITD, 48.9% of HT, 41.9% of GD, 37.1% of non-AITD (SD); lower vitamin D status in overt hypothyroid HT than other HT groups or non-AITD (SD)
Muscogiuri et al., 2016 [31]	168 elderly subjects	<50 (D)	prevalence of AIT 28% vs. 8% in D and non-D groups (SD); correlation between 25(OH) D and anti-TPO levels (*r* = −0.27) (SD)
Camurdan et al., 2012 [32]	152 children (78 recently diagnosed HT, 74 controls)	<32.5	73.1% of HT, 17.6% of controls (SD); 31.2 vs. 57.9 nmol/L (SD); inverse correlation with anti-TPO levels (*r* = −0.30) (SD)
Evliyaoğlu et al., 2015 [33]	169 Turkish children (90 HT, 79 healthy controls)	<50	71.1% of HT, 51.9% of controls (SD); 41.6 vs. 52.4 nmol/L (SD); OR 2.28 for HT risk in 25(OH)D <50 nmol/L
Metwalley et al., 2016 [34]	112 Egyptian children (56 AIT, 56 healthy, age- and sex-matched controls	<12.5 (DD) 12.5–37.5 (D) 37.5–50 (I) 50–250 (S)	vitamin D deficiency rate 71.4% of AIT, 21.4% of controls (SD); 16.2 vs. 33.9 nmol/L (SD); negative correlations between 25(OH)D and disease duration, anti-TPO, anti-Tg, and TSH (*r* = −0.676, −0.533, −0.487, −0.445, respectively) (SD)
Goswami et al., 2009 [35]	642 students, teachers and staff aged 16–60 years	<25	no association with anti-TPO positivity; weak inverse correlation between 25(OH)D and anti-TPO levels (*r* = −0.08)
Effraimidis et al., 2012 [36]	803 subjects from the Amsterdam AITD cohort	NA	no association with early stages of thyroid autoimmunity
D’Aurizio et al., 2015 [11]	100 AITD (52 HT, 48 GD), 126 healthy controls	<50 nmol/L	no difference
Yasmeh et al., 2016 [37]	97 HT, 88 healthy controls	<50 (D) 50–74.9 (I) ≥75 (S)	no association between HT and vitamin D deficiency; S 51.7% of HT vs. 31.1% controls in females (SD); 76.8 vs. 68.8 nmol/L in HT and control females (SD); correlation between 25(OH)D and anti-TPO levels (*r* = 0.436) in males (SD)
Yasuda et al., 2012 [38]	72 females (26 new onset GD, healthy controls)	<37.5 nmol/L	65.4% of GD, 32.4% of controls (SD); 35.9 vs. 42.7 nmol/L (SD); correlation between 25(OH)D and thyroid volume (*r* = −0.45) (SD)
Yasuda et al., 2013 [39]	103 females (36 non-remission GD, 18 remission GD, 49 controls)	NA	36.2 vs. 45.4 vs. 46.4 nmol/L (SD)
Zhang et al., 2015 [40]	70 GD, 70 controls	<50 nmol/L	higher vitamin D deficiency rates and lower 25(OH)D levels in anti-TSHR (+) GD than anti-TSHR (−) GD or controls (SD); inverse correlation between 25(OH)D and anti-TSHR levels in anti-TSHR (+) GD

25(OH)D, 25-hydroxyvitamin D; AITD, autoimmune thyroid disease; HT, Hashimoto’s thyroiditis; GD, Graves’ disease; SD, significant difference; anti-TPO, anti-thyroid peroxidase antibody; anti-Tg, anti-thyroglobulin antibody; NA, not available; OR, odds ratio; D, deficient; I, insufficient; S, sufficient; AIT, autoimmune thyroiditis; DD: severely deficient; TSH, thyroid stimulating hormone; anti-TSHR, anti-TSH receptor antibody.

**Table 2:** Clinical studies for an association between vitamin D status and thyroid cancer.

Sources	Study Subjects	Low Vitamin D Status (25(OH)D Level (nmol/L))	
		Criteria	Notable Findings
Roskies et al., 2012 [61]	212 patients undergoing thyroidectomy	<37.5 (D)	malignancy rate 75% vs. 37.5% in D and non-D group (RR 2.0, 95% CI 1.07–2.66) (SD)
Sahin et al., 2013 [62]	344 PTC, 116 controls	<50	70.6% of PTC, 59.3% of controls; 42.4 vs. 47.4 nmol/L (SD); association between tumor diameter and log-25(OH)D (*B* = 0.207) (SD)
Kim et al., 2014 [63]	548 females undergoing total thyroidectomy for PTC	<46.2 (median)	higher risk of T stage 3/4, LNM, lateral LNM, stage III/IV (SD); lower 25(OH)D levels in patients with a tumor size >1 cm or LNM (SD)
Stepien et al., 2010 [64]	50 TC (27 PTC, 16 FTC, 7 ATC), 34 MNG, 26 healthy controls	NA	no difference in 25(OH)D levels; lower 1,25(OH)_2_D levels in TC than controls (SD); inverse relationship between 1,25(OH)_2_D levels and tumor stage (SD)
Penna-Martinez et al., 2009 [65]	172 TC (132 PTC, 40 FTC), 321 healthy controls	<50	no difference vitamin D deficiency rates and 25(OH)D levels; higher 1,25(OH)_2_D deficiency and lower 1,25(OH)_2_D levels in TC than controls (SD)
Penna-Martinez et al., 2012 [66]	253 TC (205 PTC, 48 FTC), 302 healthy controls	<25 (DD) 25–50 (D) 50–75 (I) >75 (S)	no difference in vitamin D status and 25(OH)D levels; lower 1,25(OH)_2_D levels in TC than controls (SD); lower 25(OH)D and 1,25(OH)_2_D levels in TC patients with certain CYP24A1 haplotypes
Laney et al., 2010 [67]	69 TC (45 in remission, 24 active), 42 benign thyroid nodule patients	<75	no difference vitamin D deficiency rates and 25(OH)D levels
Jonklaas et al., 2013 [68]	65 euthyroid patients undergoing thyroidectomy	NA	no association between 25(OH)D levels and malignancy rate, stage, or other prognostic features
Lizis-Kolus et al., 2013 [69]	80 females (40 PTC, 40 HT)	NA	no association between 25(OH)D levels and malignancy rate or stage
Ahn et al., 2016 [70]	820 PTC	<24.7 24.7–32.9 33.0–44.1 44.2–110.0	no association between vitamin D status and disease aggressiveness or poor outcomes
Danilovic et al., 2016 [71]	433 patients undergoing thyroidectomy (199 TC, 234 benign nodule)	<50	no difference in vitamin D deficiency rates and 25(OH)D levels
Kim, 2016 [72]	410 patients undergoing US-guided FNA for thyroid nodules (44 TC, 366 benign)	<75	no difference in vitamin D deficiency and 25(OH)D levels; no association with cancer stage or other prognostic features
Choi et al., 2017 [73]	5186 euthyroid subjects without AITD undergoing routine health check-ups (53 TC)	<25 (D) 25–75 (I) 75–125 (S) >125 (E)	no difference in vitamin D status and 25(OH)D levels

25(OH)D, 25-hydroxyvitamin D; D, deficient; RR, relative risk; SD, significant difference; CI, confidence interval; PTC, papillary thyroid cancer; LNM, lymph node metastasis; TC, thyroid cancer; FTC, follicular thyroid cancer; ATC, anaplastic thyroid cancer; MNG, multinodular nontoxic goiter; NA, not available; 1,25(OH)_2_D, 1,25-dihydroxyvitamin D; DD, severely deficient; I, insufficient; S, sufficient; CYP24A1, 24-hydroxylase; HT, Hashimoto’s thyroiditis; US, ultrasonography; FNA, fine-needle aspiration; AITD, autoimmune thyroid disease; E, excess.
